# Evolution and Population Dynamics of Clonal Complex 152 Community-Associated Methicillin-Resistant *Staphylococcus aureus*

**DOI:** 10.1128/mSphere.00226-20

**Published:** 2020-07-01

**Authors:** Sharmin Baig, Anders Rhod Larsen, Patrícia Martins Simões, Frédéric Laurent, Thor Bech Johannesen, Berit Lilje, Anne Tristan, Frieder Schaumburg, Beverly Egyir, Ivana Cirkovic, Graeme R. Nimmo, Iris Spiliopoulou, Dominique S. Blanc, Sara Mernelius, Aina Elisabeth Fossum Moen, Michael Z. David, Paal Skytt Andersen, Marc Stegger

**Affiliations:** a Department of Bacteria, Parasites, and Fungi, Statens Serum Institut, Copenhagen, Denmark; b Centre National de Référence des Staphylocoques, Institut des Agents Infectieux, Hospices Civils de Lyon, Lyon, France; c Institute of Medical Microbiology, University Hospital Münster, Münster, Germany; d Department of Bacteriology, Noguchi Memorial Institute for Medical Research, University of Ghana, Accra, Ghana; e Institute of Microbiology and Immunology, Faculty of Medicine, University of Belgrade, Belgrade, Serbia; f Pathology Queensland Central Laboratory, Griffith University School of Medicine, Queensland Health, Brisbane, Queensland, Australia; g National Reference Laboratory for Staphylococci, Department of Microbiology, School of Medicine, University of Patras, Patras, Greece; h Service of Hospital Preventive Medicine, Lausanne University Hospital and University of Lausanne, Lausanne, Switzerland; i Laboratory Medicine, Jönköping, Region Jönköping County, Sweden; j Department of Biomedical and Clinical Sciences, Linköping University, Linköping, Sweden; k Department of Clinical Molecular Biology (EpiGen), Division of Medicine, Akershus University Hospital, Lørenskog, Norway; l Department of Microbiology and Infection Control, Akershus University Hospital, Lørenskog, Norway; m University of Oslo, Oslo, Norway; n Department of Medicine, University of Pennsylvania, Philadelphia, Pennsylvania, USA; o Department of Veterinary and Animal Sciences, University of Copenhagen, Copenhagen, Denmark; University of Minnesota

**Keywords:** CA-MRSA, CC152, MRSA, PVL, SCC*mec*, antibiotic resistance, evolution, genetics, virulence, *S. aureus*

## Abstract

Since the late 1990s, changes in the epidemiology of methicillin-resistant Staphylococcus aureus (MRSA) were recognized with the emergence of community-associated MRSA (CA-MRSA). CA-MRSA belonging to clonal complex 152 (CC152), carrying the small staphylococcal cassette chromosome *mec* (SCC*mec*) type V and encoding the Panton-Valentine leukocidin (PVL), has been observed in Europe. The aim of this study was to investigate its origin, evolution, and dissemination. Whole-genome sequencing was performed on a global collection of 149 CC152 isolates spanning 20 years (93 methicillin-susceptible S. aureus [MSSA] and 56 MRSA isolates). Core genome phylogeny, Bayesian inference, *in silico* resistance analyses, and genomic characterization were applied. Phylogenetic analysis revealed two major distinct clades, one dominated by MSSA and the other populated only by MRSA. The MSSA isolates were predominately from sub-Saharan Africa, whereas MRSA was almost exclusively from Europe. The European MRSA isolates all harbored an SCC*mec* type V (5C2&5) element, whereas other SCC*mec* elements were sporadically detected in MRSA from the otherwise MSSA-dominated clade, including SCC*mec* types IV (2B), V (5C2), and XIII (9A). In total, 93% of the studied CC152 isolates were PVL positive. Bayesian coalescent inference suggests an emergence of the European CC152-MRSA in the 1990s, while the CC152 lineage dates back to the 1970s. The CA-MRSA CC152 clone mimics the European CC80 CA-MRSA lineage by its emergence from a PVL-positive MSSA ancestor from North Africa or Europe. The CC152 lineage has acquired SCC*mec* several times, but acquisition of SCC*mec* type V (5C2&5) seems associated with expansion of MRSA CC152 in Europe.

**IMPORTANCE** Understanding the evolution of CA-MRSA is important in light of the increasing importance of this reservoir in the dissemination of MRSA. Here, we highlight the story of the CA-MRSA CC152 lineage using whole-genome sequencing on an international collection of CC152. We show that the evolution of this lineage is novel and that antibiotic usage may have the potential to select for the phage-encoded Panton-Valentine leukocidin. The diversity of the strains correlated highly to geography, with higher level of resistance observed among the European MRSA isolates. The mobility of the SCC*mec* element is mandatory for the emergence of novel MRSA lineages, and we show here distinct acquisitions, one of which is linked to the successful clone found throughout Europe today.

## Abstract (precis)

Understanding the evolution of CA-MRSA is important in light of the increasing importance of this reservoir in the dissemination of MRSA. Here, we highlight the story of the CA-MRSA CC152 lineage using whole-genome sequencing on an international collection of CC152. We show that the evolution of this lineage is novel and that antibiotic usage may have the potential to select for the phage-encoded Panton-Valentine leukocidin. The diversity of the strains correlated highly to geography, with higher level of resistance observed among the European MRSA isolates. The mobility of the SCC*mec* element is mandatory for the emergence of novel MRSA lineages, and we show here distinct acquisitions, one of which is linked to the successful clone found throughout Europe today.

## INTRODUCTION

In the late 1990s, a remarkable change in the epidemiology of Staphylococcus aureus was observed with the emergence of methicillin-resistant S. aureus (MRSA) in the community (1, 2). Today, multiple lineages of community-associated MRSA (CA-MRSA) are observed worldwide (3), and the evolutionary history of some of these has been described in detail using whole-genome sequencing on global collections of isolates (4–7). As common characteristics, CA-MRSA clones generally are less resistant to antibiotics than health care-associated MRSA (HA-MRSA), harbor smaller staphylococcal cassette chromosome *mec* (SCC*mec*) type IV or V elements, and often express the Panton-Valentine leukocidin (PVL) toxin encoded by the prophage genes *lukS*/*F*-PV. However, the clinical epidemiological distinction between HA- and CA-MRSA has started to dissipate as CA-MRSA lineages, in particular USA300, have expanded their niches into hospitals (8, 9). CA-MRSA clones have been observed to be geographically restricted (10), with the European CA-MRSA as one example. This lineage, belonging to clonal complex 80 (CC80), was derived from a PVL-positive methicillin-susceptible S. aureus (MSSA) ancestor from sub-Saharan Africa and became a dominant CA-MRSA clone in Europe, Northern Africa, and the Middle East in the 1990s and 2000s (5). Other well-characterized CA-MRSA lineages that also have shown intercontinental transmission include USA300, where genome analyses point to a European ancestor prior to acquisition of PVL and SCC*mec* type IV in the United States (6), and the multidrug-resistant and PVL-positive Bengal Bay clone (MLST sequence type 772 [ST772]) that has spread globally with an origin in community- and health care-associated environments in India (4).

The different CA-MRSA lineages observed worldwide have shown different geographical origins and dissemination patterns and with key genetic acquisitions strongly associated with their expansion. The S. aureus lineage CC152 has been linked to community cases throughout Europe and in particular with strong epidemiological links to the Balkan region and encodes key CA-MRSA genetic features such as PVL and carriage of a small SCC*mec* type V element (3, 11). CC152 PVL-positive MSSA isolates have been reported in the Canary Islands and in sub-Saharan Africa and was found as a highly dominant nasal carrier clone in Mali (11). In the present study, an international collection of CC152 MSSA and MRSA isolates was investigated to understand the origin, evolution, and dissemination of the lineage.

## RESULTS

### Strain collection and genome sequencing.

**FIG 1:**
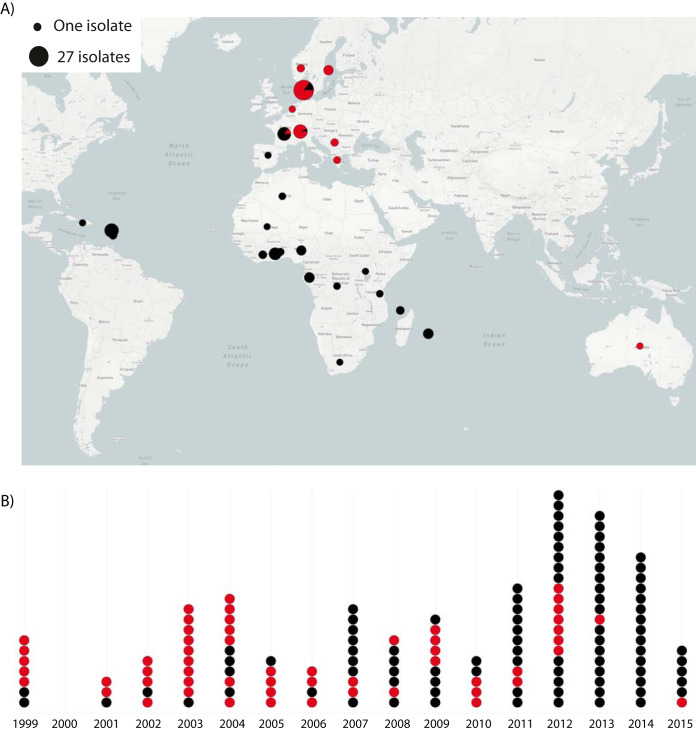
Geographical and temporal distribution of *S. aureus* CC152 isolates. Black and red dots indicate MSSA (*n* = 93) and MRSA (*n* = 56) isolates, respectively. (A) Geographical distribution of the CC152 isolates from 28 different countries in Europe, Africa, Australia, and the Caribbean. The dots are scaled according to the prevalence as indicated. (B) Timeline showing the sampling years of the isolates (1999 to 2015).

This study included 149 CC152 S. aureus isolates (93 MSSA and 56 MRSA) sampled between 1999 to 2015 in 28 different countries in Europe, Africa, Australia, and the Caribbean Islands (Fig. 1; see also Table S1 in the supplemental material). Of these, 31 were selected and obtained for genome sequencing based on the MRSA TypeCat database to expand the temporal and geographical diversity of the collection and 10 genomes were obtained from NCBI’s RefSeq database.

10.1128/mSphere.00226-20.5 TABLE S1 Overview of CC152 study collection including geographical origin, isolation year, PVL, SCC*mec* type, MLST profiles, *spa* repeats, and resistance genes. Download Table S1, XLSX file, 0.02 MB. Copyright © 2020 Baig et al. 2020 Baig et al. This content is distributed under the terms of the Creative Commons Attribution 4.0 International license.

The majority of the CC152 isolates belonged to ST152 (*n* = 139; 93%); of the remaining 10 isolates (seven MSSA and three MRSA), 9 were single-locus variants of ST152 (four ST1518, one ST1831, and four different new STs), whereas another single isolate was a double-locus variant of ST152 (new ST; see Table S1). Twenty-two different *spa* types were identified, with t355 being the most common (*n* = 98; 65.8%; see Table S1). The isolates were sequenced to an average depth of ∼120-fold.

### Phylogenetic relatedness and geography.

**FIG 2:**
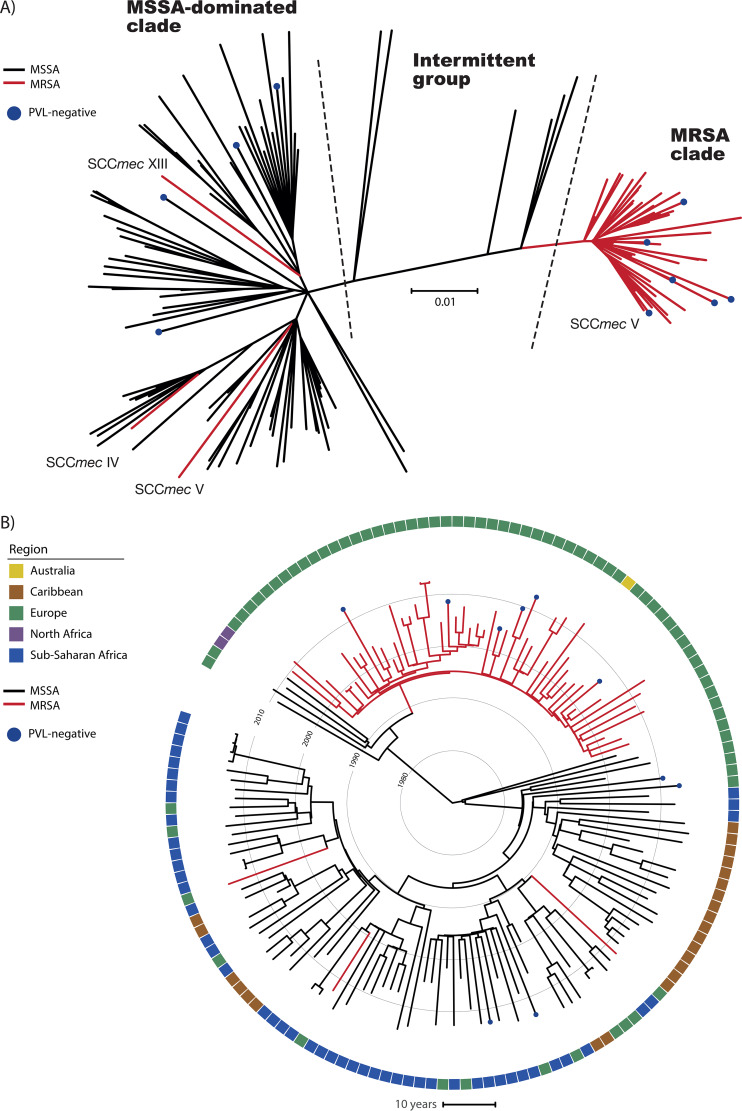
Temporal and geographical relatedness of the *S. aureus* CC152 lineage. The phylogenies are based on 5,149 SNPs identified in 82% of the reference chromosome. (A) Unrooted phylogeny of the 149 CC152 isolates. (B) Time-based tree of the 149 CC152 isolates. The black and red branches indicate the MSSA and MRSA isolates, respectively. All isolates are PVL positive except when blue dots are present. The colors in the outer ring represent the continental origins of the isolates as follows: Australia (yellow), Caribbean (brown), Europe (green), North Africa (purple), and sub-Saharan Africa (blue).

A total of 5,189 single nucleotide polymorphisms (SNPs) was detected within 82% (∼2.19 Mb) of the detected CC152 core genome among all 149 isolates. Five different strains, which were used for rooting the tree, all revealed identical rooting and overall topology (data not shown). The rooted phylogeny was based on 5,149 SNPs after purging of eight putative recombinant regions spanning between three and 15 nucleotides (see Fig. S1 in the supplemental material). An unrooted CC152 phylogeny (Fig. 2A) showed mainly two distinct clades, one contained primarily MSSA isolates (MSSA-dominated clade, Fig. 2A), and the other contained only MRSA isolates (MRSA clade, Fig. 2A). By rooting the phylogeny (Fig. 2B) and population with geographical data, the MRSA clade was linked almost exclusively to Europe, while the other more diverse clade contained mainly isolates from sub-Saharan Africa and the Caribbean but also some with a European origin. All major clades had high posterior bootstrap values (Fig. S3).

10.1128/mSphere.00226-20.1 FIG S1 Rooted maximum-likelihood phylogeny of the 149 CC152 isolates based on 5,149 SNPs. The colored branches indicate MSSA (black) and MRSA (red) isolates. The blue dots indicate PVL-negative isolates, while the outer circles show the presence (black) or absence of individual resistance genes. The scale bar indicates substitutions per site. Download FIG S1, EPS file, 2.7 MB. Copyright © 2020 Baig et al. 2020 Baig et al. This content is distributed under the terms of the Creative Commons Attribution 4.0 International license.

### SCC*mec* acquisition and diversity.

The MRSA clade contained the majority of MRSA isolates (53/56, 95%), which all carried SCC*mec* type V (5C2&5), indicating a single acquisition of the SCC*mec* element (Fig. 2A). Four isolates had a deletion of a smaller region containing the *ccrC1* gene. The remaining three MRSA isolates (55-99-35, 55-99-40, and 55-99-44) in the collection were unrelated and clustered in the MSSA-dominated clade. These isolates carried three different SCC*mec* elements: a type V (5C2), a type IV (2B), and a type XIII (9A).

### Prevalence of the Panton-Valentine leukocidin-encoding φSa2 prophage.

Among the CC152 isolates, the majority (*n* = 139, 93%,) were PVL positive and carried *lukF*/*S*-PV genes in the φSa2 prophage. Sequence analysis of the diversity of the φSa2 PVL-prophage (Fig. S2) among CC152 and other φSa2 positive CA-MRSA lineages clearly support a single ancestral acquisition of the φSa2 prophage in CC152. The PVL-negative isolates (*n* = 10) were generally scattered across the tree, indicating independent loss of the prophage (Fig. 2).

10.1128/mSphere.00226-20.2 FIG S2 Maximum-likelihood phylogeny based on purged SNPs (*n* = 874) of the PVL-encoding prophage of the 139 PVL-positive CC152 isolates, including representatives of major CA-MRSA lineages (CC1, CC8, CC30, CC59, CC80, and CC93). The scale bar indicates substitutions per site. Download FIG S2, EPS file, 1.3 MB. Copyright © 2020 Baig et al. 2020 Baig et al. This content is distributed under the terms of the Creative Commons Attribution 4.0 International license.

10.1128/mSphere.00226-20.3 FIG S3 Time-based tree of S. aureus CC152 with bootstrap values. The phylogeny is based on 5,149 SNPs identified among 82% of the reference chromosome as in Fig. 2B. The black and red branches indicate the MSSA and MRSA isolates, respectively. The PVL-negative isolates are indicated with blue dots. The outer line represents the regional origins of the isolates as follows: Australia (yellow), Caribbean (brown), Europe (green), North Africa (purple), and sub-Saharan Africa (blue). Posterior bootstrap values of ≥85 are represented as grey dots. The sample IDs for each isolate are shown. The scale bar indicates time in years. Download FIG S3, EPS file, 2.6 MB. Copyright © 2020 Baig et al. 2020 Baig et al. This content is distributed under the terms of the Creative Commons Attribution 4.0 International license.

### Coalescent analysis of CC152.

The best model for the Bayesian analysis was the general time reversible (GTR) model under the random local clock and the Bayesian Skyline prior with a >10 Bayes factor. The analysis revealed that the time to most recent common ancestor (TMRCA) of the CC152 lineage dated to ∼1970 (95% highest posterior densities [HPDs], 1964 to 1974), and with the emergence of the MRSA clade ∼25 years later (95% HPDs, 1993 to 1995) based on the included strain collection. The time tree shared a highly similar tree topology (Fig. 2B) with the rooted maximum-likelihood phylogeny (Fig. S1). The Skyline plot (Fig. S4) shows a large increase in effective population size coinciding with the acquisition of the SCC*mec* element in the monophyletic MRSA clade. The time-based tree (Fig. 2B) further supports the hypothesis that the ancestral population of CC152 was PVL positive. All MRSA isolates were from Europe except for one isolate from Australia, regardless of the position in the phylogeny (Fig. 2A). The majority of the MSSA isolates formed a large clade of isolates from Europe, sub-Saharan Africa, and the Caribbean. Six MSSA isolates (intermittent group, Fig. 2A) clustered between the MSSA-dominated clade and the MRSA clade with four from Europe and two from North Africa. Combined, the analyses point to an origin of CC152 lineage in North Africa or in Europe.

10.1128/mSphere.00226-20.4 FIG S4 Effective population size through time of the CC152 lineage. A long stationary phase is observed until 1990, where an increase was observed in the effective CC152 population size, which overlaps with the SCC*mec* acquisition around 1995 and the emergence of the successful CC152 MRSA lineage. Download FIG S4, EPS file, 2.9 MB. Copyright © 2020 Baig et al. 2020 Baig et al. This content is distributed under the terms of the Creative Commons Attribution 4.0 International license.

### Resistance determinants and genetic context.

Among the 149 isolates, 14 different resistance genes were detected (Table 1 and Fig. S1). The predominant resistance determinant among the 149 CC152 isolates was *blaZ* (*n* = 137, 92%), whereas the aminoglycoside resistance gene *aac(6′)-aph(2″)* was almost exclusively found in the MRSA clade (*n* = 51, 96%) but rarely found outside this clade (*n* = 2, 2%). Analysis of the predominant SCC*mec* type V (5C&5C2) showed the *aac(6′)-aph(2″)* gene to be part of the J2 region of the element in 93% of these isolates, including all isolates in the MRSA clade. The aminoglycoside resistance gene *aadD* was only found in 19% of isolates (*n* = 10/96) in the MRSA clade. However, *dfrG* conferring resistance to trimethoprim was found in 19% (*n* = 18/96) of the MSSA isolates found to reside in two distinct monophyletic groups: one included 10 isolates where *dfrG* resided in the PVL-encoding prophage φSa2, whereas in the other monophyletic group of 8 isolates no prophage or other identifiable genetic element was identified in the vicinity of the resistance gene (data not shown). No isolates carried known *fusA* mutations, and only a single MRSA isolates carried the *fusB* gene linked to fusidic acid resistance. Carriage of the tetracycline resistance determinant *tet*(K) was found throughout the phylogeny, with 19% (*n* = 10/49) of the isolates positive in the MRSA clade and 29% (*n* = 28/96) among the remaining isolates, all located on a pT181 plasmid.

**TABLE 1:** Overview of different resistance genes, showing the antibiotic class and number of isolates for each resistance gene according to the phylogenetic clustering

Resistance gene	Antibiotic class	No. (%) of isolates	
		MRSA clade (*n* = 53)	MSSA-dominated clade (*n* = 96)
*tet*(K)	Tetracycline	10 (19)	28 (29)
*cat*(pC233)	Phenicol	6 (11)	0
*cat*(pC221)	Phenicol	1 (2)	5 (5)
*blaZ*	Beta-lactam	49 (92)	88 (92)
*mecA*, SCC*mec* type	Beta-lactam	53 (100), V(5C2&5)^*a*^	3 (3), IV, V(5C2), XIII
*aac(6′)-aph(2″)*	Aminoglycoside	2 (2)	51 (96)
*aadD*	Aminoglycoside	0	10 (19)
*aph(3′)-III*	Aminoglycoside	1 (1)	0
*str*	Aminoglycoside	3 (3)	0
*dfrG*	Trimethoprim	18 (19)	0
*fusB*	Fusidic acid	1 (1)	0
*msr*(A)	Macrolide, lincosamide, streptogramin B	1 (1)	0
*lnu*(A)	Macrolide, lincosamide, streptogramin B	0	8 (15)
*erm*(C)	Macrolide, lincosamide, streptogramin B	2 (2)	2 (4)

a Four isolates only had one *ccrC1* gene equivalent to an SCC*mec* type V(5C2).

## DISCUSSION

The S. aureus CC152 lineage has been reported as a PVL-positive CA-MRSA that has only rarely been reported outside the European continent. Conversely, PVL-positive CC152 MSSA isolates have been associated with the African continent and the Caribbean and less often in Europe (8, 11). Thus, the evolution of CC152 resembles in many ways the genotypic and spatial characteristics of the European CC80 CA-MRSA clone (5). Here, we analyzed a collection covering 28 countries and spanning from the earliest reported isolates in 1999 until 2015 to understand the evolution of the CC152 lineage.

The data in this study, combining molecular analyses in conjunction with temporal and spatial information, indicate that the CA-MRSA CC152 clone originated north of the Sahara from a PVL-positive MSSA ancestor. Using both phylogenetic approaches with the available data, deep branches near the root all point to European or Northern African MSSA isolates. Specifically, a small cluster of three isolates, including two North African (Algerian) and a single European (French) isolate, clustered ancestral to the monophyletic European MRSA clade (Fig. 2B). However, since no epidemiological information about these three isolates were available and there is a cultural and geographical proximity between France and Northern Africa, our data do not allow us to infer the precise geographical origin of the MRSA ancestor. The other major clade identified in the phylogeny contained mostly MSSA isolates of sub-Saharan African and Caribbean isolates. Ruimy et al. proposed that, similarly to the European CC80 CA-MRSA clone, the CC152 lineage originated in sub-Saharan Africa and spread to Europe and subsequently acquired the SCC*mec* element (11). However, the results of our genome-based analyses do not support such a sub-Saharan origin.

Multiple CA-MRSA lineages emerged globally in the 1990s and are generally predominantly found in specific geographical regions (10) exemplified by the European CA-MRSA CC80 clone, USA300 (5, 6, 12), and now the CC152 CA-MRSA clone. In both the ST8 and the CC80 lineages, SCC*mec* elements were acquired on multiple occasions (6, 13, 14); however, successful clones, including both the North and South American variants of USA300 and the European CA-MRSA, are all associated with single acquisitions of an SCC*mec* element. A similar pattern is observed for the CC152 lineage, where multiple acquisitions of distinct SCC*mec* elements, including an SCC*mec* element type XIII (15), are evident, but only the acquisition of SCC*mec* type V (5C2&5) is associated with its expansion in Europe. Another key characteristic of the CA-MRSA lineages is the presence of PVL-encoding genes; both CC80 and CC152 have ancestral PVL acquisitions, whereas the CC8 lineage has an ancestral PVL-negative population (5, 6, 11). The finding here of *dfrG*, a determinant for trimethoprim resistance, in the φSa2 prophage is interesting since trimethoprim resistance is common in S. aureus from sub-Saharan Africa. This has previously been linked to the prophylactic use of trimethoprim in HIV-infected persons (16, 17), and thus trimethoprim may select for carriage of such PVL-positive isolates.

In general, CA-MRSA has been categorized as carrying fewer resistance genes compared to HA-MRSA (18); however, the CC152 MRSA isolates in addition to *mecA* and *blaZ* also harbor an aminoglycoside resistance gene [*aac(6′)-aph(2″)*] found to reside in the SCC*mec* element for 95% (53/56) of the MRSA isolates. Since aminoglycoside antibiotics are mainly used in hospitals, (19), this may explain why the CC152 MRSA clone spread and thrived in hospitals, as well as in the community (20). Overall, a greater number of resistance genes was observed among the MRSA than among MSSA isolates, but this could be the result of sampling bias. Interestingly, only a single MRSA isolate was genotypically resistant to fusidic acid due to the carriage of *fusB*, and investigations into resistance-related mutations in *fusA* showed the absence of any such variants. This is striking compared to the prevalence and speculated importance of fusidic acid resistance observed in the European CC80 CA-MRSA clone (5).

Of the sub-Saharan cases with epidemiological data, 15% of the CC152 MSSA isolates were reported from bacteremia cases, which likely reflects its high prevalence as a carrier clone in sub-Saharan Africa rather than being an indication of increased virulence potential. The results of this study show an overall low level of resistance, restricted geographical spread, the harboring of smaller SCC*mec* elements, and carriage of genes encoding PVL in CC152 and hence a resemblance to other CA-MRSA clones.

In conclusion, we demonstrate that multiple SCC*mec* acquisitions have occurred in the CC152 lineage; however, only one is linked to the successful clone now found throughout Europe. Our analyses support a unique evolutionary path of this PVL-positive clone that most likely indicates a European or North African ancestral origin with the emergence of the CA-MRSA clone around the 1990s, similar to findings for other investigated CA-MRSA lineages.

## MATERIALS AND METHODS

### Bacterial isolates.

A total of 149 S. aureus CC152 isolates, including 93 MSSA and 56 MRSA isolates, sampled in Australia (*n* = 1), the Caribbean (*n* = 24), Europe (*n* = 73), northern Africa (*n* = 2), and sub-Saharan Africa (*n* = 49) between 1999 and 2015 were included in this study. To obtain representative temporal and spatial collection of CC152 genomes, isolates and publicly available genomic data were selected and obtained from national reference collections, NCBI’s Reference Sequence Database (https://www.ncbi.nlm.nih.gov/refseq/) and the MRSA TypeCat database (21) that contains a systematic review of S. aureus in all indexed literature with information on genotypic data published between 2000 and 2015 (see Table S1). Microreact (https://microreact.org) v5.112.3 was used to visualize the geographical and temporal distribution of the CC152 isolates (Fig. 1).

### Ethics.

All isolates and epidemiological information were obtained either based on previously published work or by local regulations that allow use for research.

### Genome sequencing.

In this study, we performed whole-genome sequencing on 139 CC152 isolates using a Nextera XT DNA Library Prep kit (Illumina) generated libraries to obtain pair-end data on Illumina’s NextSeq and MiSeq platforms with 300- or 500-cycle kits. All generated sequence reads are deposited at the European Nucleotide Archive (ENA; https://www.ebi.ac.uk/ena) under study accession no. PRJEB36544. The remaining 10 genomes were downloaded from the NCBI (Table S1).

### Phylogenetic analysis.

SNPs were detected in the core genome using the Northern Arizona SNP pipeline (NASP [22]) by aligning Illumina reads against the 2.67-Mbp chromosome of S. aureus ST152 MSSA strain SA17_S6 (GenBank accession number CP010941). Positions with a <10-fold sequencing depth and/or <90% unambiguous base calls were removed. Putative signs of recombination, defined as if more than three consecutive SNPs in individual or clusters of isolates were observed in the resulting SNP matrix, were removed as previously described (5). Relatedness was investigated using the maximum-likelihood algorithm as implemented in PhyML v3.0 (23, 24), using smart model selection with the Bayesian information criterion with 100 bootstrap replicates and with subtree-pruning and -grafting rearrangements for improved tree structures. For rooting, the chromosome of five unrelated S. aureus strains belonging to ST8, ST30, ST45, ST80, and ST398 (GenBank accession numbers CP000730, BX571856, NC_021554, CP003194, and AM990992, respectively) were used individually. The phylogenetic trees were visualized using iTOL v4.2.3 (25).

### Coalescent analyses.

Bayesian analyses were used to investigate the TMRCA method using BEAST v1.8.4 (26). BEAST was performed using the GTR and Hasegawa-Kishino-Yano (HKY) substitution models combined with both strict and random local clock under different coalescent tree priors (Bayesian Skyline, constant population, and exponential growth). Evaluation of the model combinations was performed by Tracer v1.7.1 (http://tree.bio.ed.ac.uk/software/tracer/) using Bayes factor (27). All Markov chain Monte Carlo analyses were run twice with chain lengths of 200 million with sampling every 20,000th generation and using a burn-in set at 10%. A time tree was obtained using the maximum sum clade credibility topology with TreeAnnotator v2.5.2 from the BEAST package and visualized using iTOL.

### Genetic typing.

Whole-genome sequences were *de novo* assembled using SPAdes v3.13.1 (28). Assemblies were used for *in silico* multilocus sequence typing (MLST) (28, 29) passed through spaTyper v1.0 (30), ResFinder v2.1 (31), and SCC*mec*Finder v1. (32) for the determination of *spa* types, resistance genes, and SCC*mec* characterization, respectively, at the Center for Genomic Epidemiology (https://cge.cbs.dtu.dk). Furthermore, CLCbio’s Genomics Workbench v9 (Qiagen, Aarhus, Denmark) was used for additional investigations of resistance genes and SCC*mec* characterization. Similarly, detection of point mutations related to fusidic acid resistance was performed using BLASTN searches across the collection using *fusA* extracted from the ST152 reference genome (GenBank accession no. CP010941). The draft genomes were analyzed for the presence of PVL-encoding genes *lukF/S*-PV and for intradiversity of the associated φSa2 prophage within the CC152 collection and other S. aureus clonal lineages. The full-length φSa2 prophage in the ST152 reference was identified using the PHAge Search Tool Enhanced Release (PHASTER) tool (http://phaster.ca/). The identified φSa2 prophage in ST152 reference genome was used as reference in a SNP-based analysis, including all PVL-positive CC152 isolates together with genomes of other CA-MRSA PVL-positive lineages (CC1, CC8, CC30, CC59, CC80, and CC93; GenBank accession no. NC_003923, CP000730, CP002110, NC_016928, NC_017351, and CP002114, respectively). The SNP alignment of the prophage was purged for putative recombination, and a phylogeny was constructed with PhyML v3.0 as previously described.

### Data availability.

All generated sequence reads are deposited at the ENA (https://www.ebi.ac.uk/ena) under study accession no. PRJEB36544.
